# Supplementary material for: Instructed knowledge shapes feedback-driven aversive learning in striatum and orbitofrontal cortex, but not the amygdala
Source: eLife. 2016 May 12;5:e15192. doi: 10.7554/eLife.15192 (PMC4907691; doi:10.7554/eLife.15192)
Supplement: Figure 3—figure supplement 1—source data 1. — This table presents brain regions that correlate with feedback-driven EV (derived from the across-subjects model fit to Uninstructed Group learners) within Uninstructed Group learners (n = 20). Results are whole-brain FDR-corrected (q < 0.05) and clusters are defined based on contiguity with voxels at uncorrected p<0.001 and p<0.01. DOI: http://dx.doi.org/10.7554/eLife.15192.009 [file elife-15192-fig3-figsupp1-data1.docx]

*Figure 3 – figure supplement 1 - Source data 1. Neural correlates of feedback-driven expected value (EV): Uninstructed Group Learners (n = 20)^a^*

| **Contrast** | **Region** | **x** | **y** | **z** | **Number of voxels** | **Robust regression intercept** |
| --- | --- | --- | --- | --- | --- | --- |
| *Positive correlation with feedback-driven EV* | R Cerebelum VII | 44 | -60 | -54 | 31 | 10.61 |
|  | L Fusiform Gyrus | -32 | -12 | -42 | 30 | 12.32 |
|  | L Cerebelum VII | -32 | -34 | -40 | 15 | 12.18 |
|  | L Pons | -12 | -26 | -36 | 10 | 9.71 |
|  | R Inferior Temporal Gyrus | 50 | -18 | -32 | 15 | 9.2 |
|  | R Superior Orbital Gyrus | 14 | 38 | -24 | 48 | 9.01 |
|  | L Amygdala (CM) | -22 | -2 | -18 | 114 | 13.71 |
|  | R ParaHippocampal Gyrus/ Entorhinal Cortex | 22 | -2 | -30 | 14 | 9.37 |
|  | L IFG p. Opercularis | -46 | 14 | 20 | 2453 | 20.54 |
|  | L Fusiform Gyrus | -38 | -26 | -26 | 38 | 12.86 |
|  | R IFG p. Orbitalis | 28 | 14 | -24 | 85 | 15.69 |
|  | L Fusiform Gyrus | -28 | -78 | -16 | 241 | 12.92 |
|  | L Fusiform Gyrus ( Area hOc4v [V4(v)]) | -44 | -52 | -20 | 227 | 12.67 |
|  | L Superior Orbital Gyrus (mOFC) | -18 | 42 | -24 | 15 | 12.28 |
|  | R Inferior Temporal Gyrus | 54 | -46 | -20 | 66 | 10.43 |
|  | R Fusiform Gyrus | 42 | -44 | -22 | 42 | 10.8 |
|  | R Fusiform Gyrus | 36 | -60 | -12 | 590 | 11.9 |
|  | R Amygdala (SF) / Hippocampus | 20 | -4 | -18 | 74 | 11.74 |
|  | R Middle Temporal Gyrus | 72 | -28 | -16 | 27 | 13.06 |
|  | R IFG p. Triangularis | 48 | 18 | 22 | 2009 | 15.7 |
|  | R Inferior Temporal Gyrus / Area hOc4la | 54 | -74 | -8 | 37 | 9.59 |
|  | R Lingual Gyrus/ Area hOc3v [V3v] | 14 | -70 | -6 | 43 | 15.37 |
|  | L Inferior Occipital Gyrus | -50 | -64 | -12 | 11 | 9.3 |
|  | L Lingual Gyrus/ Area hOc1 [V1] | -16 | -54 | -4 | 234 | 16.56 |
|  | R Middle Temporal Gyrus | 56 | -46 | 2 | 904 | 16.06 |
|  | L Inferior Occipital Gyrus/ Area hOc4la | -48 | -78 | -8 | 47 | 11.09 |
|  | R Calcarine Gyrus/ Area hOc1 [V1] | 14 | -100 | 2 | 68 | 9.26 |
|  | R Lingual Gyrus/ Area hOc1 [V1] | 4 | -80 | -2 | 89 | 13.24 |
|  | L Caudate Nucleus / Nucleus Accumbens (NAcc) | -8 | 10 | -4 | 98 | 11.64 |
|  | R Caudate Nucleus | 12 | 6 | 10 | 214 | 19.46 |
|  | L Middle Temporal Gyrus | -56 | -46 | 10 | 826 | 20.72 |
|  | NAcc/ Bed Nucleus of the Stria Terminalis (BNST) | 6 | 2 | 2 | 39 | 9.79 |
|  | L Thalamus | -28 | -30 | 2 | 12 | 9.49 |
|  | L Caudate Nucleus | -12 | 6 | 10 | 200 | 10.89 |
|  | R Calcarine Gyrus/ Area hOc1 [V1] | 18 | -58 | 4 | 43 | 9.43 |
|  | R Cuneus/ Area hOc2 [V2] | 6 | -94 | 14 | 316 | 14.49 |
|  | L Middle Occipital Gyrus | -46 | -82 | 8 | 31 | 11.28 |
|  | L Thalamus | -4 | -10 | 10 | 27 | 13.18 |
|  | L Caudate Nucleus (caudate tail), contiguous with L Middle Insula | -28 | -6 | 20 | 90 | 10.38 |
|  | L Middle Occipital Gyrus | -30 | -86 | 22 | 173 | 11.94 |
|  | L Cuneus/ Area hOc2 [V2] | 2 | -76 | 18 | 39 | 9.57 |
|  | R Rolandic Operculum/ Area OP4 [PV] | 62 | -12 | 14 | 13 | 13.9 |
|  | R Middle Occipital Gyrus | 38 | -84 | 26 | 156 | 11.56 |
|  | R Superior Occipital Gyrus | 22 | -72 | 18 | 22 | 13.11 |
|  | L Superior Medial Gyrus (DMPFC) | 2 | 30 | 40 | 756 | 11.42 |
|  | L Middle Occipital Gyrus | -28 | -70 | 28 | 76 | 10.02 |
|  | L Postcentral Gyrus | -66 | -10 | 26 | 41 | 9.09 |
|  | L Superior Frontal Gyrus (DMPFC) | -14 | 54 | 30 | 332 | 11.31 |
|  | R Middle Frontal Gyrus (DMPFC) | 30 | 62 | 24 | 72 | 12.87 |
|  | L Cuneus/ Area hOc3d [V3d] | -8 | -80 | 24 | 12 | 9.19 |
|  | R Superior Medial Gyrus (DMPFC) | 10 | 62 | 28 | 28 | 8.9 |
|  | L SupraMarginal Gyrus Area PFt (IPL) | -62 | -24 | 32 | 213 | 17.82 |
|  | R Cuneus/ Area hOc3d [V3d] | 6 | -82 | 32 | 36 | 9.09 |
|  | R Middle Occipital Gyrus | 38 | -70 | 30 | 37 | 9.67 |
|  | L Superior Frontal Gyrus (DMPFC) | -24 | 30 | 34 | 58 | 9.89 |
|  | L Inferior Parietal Lobule / Area PFt (IPL) | -52 | -36 | 42 | 22 | 10.49 |
|  | R Superior Medial Gyrus (DMPFC) | 10 | 56 | 44 | 19 | 10.19 |
| *Negative correlation with feedback-driven EV* | L Rectal Gyrus (mOFC) | -4 | 26 | -20 | 45 | 18.41 |
|  | R Mid Orbital Gyrus/ Area Fp2 (MPFC) | 10 | 54 | -4 | 22 | 10.78 |

^a^. This table presents brain regions that correlate with feedback-driven EV (derived from the across-subjects model fit to Uninstructed Group learners) within Uninstructed Group learners (n = 20). Results are whole-brain FDR-corrected (q < .05) and clusters are defined based on contiguity with voxels at uncorrected p < .001 and p < .01.
