# Supplementary material for: Instructed knowledge shapes feedback-driven aversive learning in striatum and orbitofrontal cortex, but not the amygdala
Source: eLife. 2016 May 12;5:e15192. doi: 10.7554/eLife.15192 (PMC4907691; doi:10.7554/eLife.15192)
Supplement: Figure 3—figure supplement 1—source data 2. — This table presents brain regions that correlate with feedback-driven EV (derived from the across-subjects model fit to Uninstructed Group learners) within the entire Uninstructed Group (n = 38). Results are whole-brain FDR-corrected (q < 0.05) and clusters are defined based on contiguity with voxels at uncorrected p<0.001 and p<0.01. DOI: http://dx.doi.org/10.7554/eLife.15192.010 [file elife-15192-fig3-figsupp1-data2.docx]

*Figure 3 – figure supplement 1 - Source data 2. Neural correlates of feedback-driven EV: Entire Uninstructed Group (n = 38)^a^*

| **Contrast** | **Region** | **x** | **y** | **z** | **Number of voxels** | **Robust regression intercept** |
| --- | --- | --- | --- | --- | --- | --- |
| Positive correlation with feedback-driven EV | L Cerebelum VIII | -30 | -68 | -56 | 14 | 7.93 |
|  | L Cerebelum VIII | -26 | -42 | -52 | 10 | 6.7 |
|  | L Fusiform Gyrus | -26 | -6 | -44 | 112 | 13.11 |
|  | R Inferior Temporal Gyrus | 38 | -6 | -46 | 27 | 7.2 |
|  | R Cerebelum X | 24 | -34 | -42 | 29 | 12.54 |
|  | L Cerebelum X | -26 | -32 | -40 | 32 | 10.89 |
|  | R Inferior Temporal Gyrus | 48 | 0 | -42 | 17 | 8.24 |
|  | R Cerebelum Crus 2 | 52 | -70 | -40 | 25 | 8.13 |
|  | L Inferior Temporal Gyrus | -46 | -16 | -34 | 14 | 6.8 |
|  | R Amygdala (LB)/ ParaHippocampal Gyrus | 22 | 2 | -22 | 273 | 16.99 |
|  | R Superior Orbital Gyrus (mOFC) | 12 | 40 | -30 | 103 | 9.73 |
|  | L Amygdala (LB) | -20 | -2 | -22 | 160 | 14.29 |
|  | L IFG p. Orbitalis | -36 | 26 | -16 | 724 | 12.57 |
|  | L Calcarine Gyrus | -12 | -56 | 4 | 14245 | 18.15 |
|  | R ParaHippocampal Gyrus | 30 | -24 | -26 | 15 | 9.51 |
|  | R Rectal Gyrus (mOFC) | 2 | 60 | -22 | 59 | 10.54 |
|  | L IFG p. Orbitalis (latOFC) | -44 | 50 | -14 | 42 | 10.37 |
|  | R IFG p. Opercularis (DLPFC) | 48 | 16 | 26 | 3170 | 21.17 |
|  | R Middle Temporal Gyrus | 60 | -24 | -10 | 11 | 7.55 |
|  | Thal: Premotor | 10 | -22 | -4 | 11 | 7.5 |
|  | L Superior Temporal Gyrus/ Area TE 3 | -58 | -4 | -4 | 13 | 7.55 |
|  | Caudate, Bilateral (contiguous) | 6 | 2 | 6 | 399 | 17.54 |
|  | Area TE 3 | -66 | -8 | 6 | 27 | 8.17 |
|  | R Insula Lobe | 36 | -12 | 4 | 15 | 7.47 |
|  | L Caudate Nucleus | -12 | 12 | 10 | 53 | 9.2 |
|  | L Rolandic Operculum (dpIns) | -40 | -8 | 12 | 17 | 7.83 |
|  | R Thalamus | 22 | -24 | 12 | 18 | 9 |
|  | L Middle Frontal Gyrus (DLPFC) | -40 | 58 | 14 | 17 | 8.13 |
|  | L Superior Medial Gyrus (DMPFC) | -4 | 40 | 40 | 1702 | 12.58 |
|  | Thal: Temporal | 16 | -22 | 20 | 22 | 6.77 |
|  | R MCC (dACC) | 2 | 0 | 30 | 175 | 9.91 |
|  | R Middle Frontal Gyrus (DLPFC) | 36 | 54 | 26 | 290 | 12.53 |
|  | R SupraMarginal Gyrus/ Area PFm (IPL) | 64 | -50 | 30 | 92 | 8.39 |
|  | L Middle Frontal Gyrus (DMPFC) | -28 | 30 | 34 | 55 | 10.29 |
|  | R SupraMarginal Gyrus/ Area PF (IPL) | 62 | -26 | 42 | 154 | 10.66 |
|  | L Middle Frontal Gyrus (DMPFC) | -36 | 38 | 34 | 25 | 7.78 |
|  | L MCC | -10 | 10 | 38 | 21 | 9.15 |
|  | R SupraMarginal Gyrus/ Area 7PC (SPL) | 42 | -36 | 42 | 22 | 6.85 |
|  | L Inferior Parietal Lobule / Area PFt (IPL) | -52 | -36 | 42 | 36 | 9.96 |
|  | L Superior Frontal Gyrus (DMPFC) | -20 | 28 | 46 | 22 | 8.31 |
|  | L Inferior Parietal Lobule / Area hIP1 (IPS) | -38 | -44 | 46 | 15 | 8.08 |
| *Negative correlation with feedback-driven EV* | L Cerebelum IX | -2 | -54 | -54 | 12 | 7.12 |
|  | L Cerebelum IX | -6 | -54 | -46 | 11 | 7.27 |
|  | R Cerebelum Crus 2 | 8 | -92 | -34 | 30 | 8.33 |
|  | Lobule VIIa crusI Hem | 36 | -90 | -32 | 14 | 9.27 |
|  | R Cerebelum Crus 1 | 46 | -82 | -28 | 31 | 9.11 |
|  | R Middle Temporal Gyrus | 64 | -8 | -20 | 28 | 16.79 |
|  | L ParaHippocampal Gyrus/ Subiculum | -24 | -26 | -16 | 26 | 8.84 |
|  | L Mid Orbital Gyrus/ Area s24 (mOFC/VMPFC) | -4 | 24 | -14 | 27 | 8.18 |
|  | L Superior Orbital Gyrus (mOFC) | -14 | 44 | -14 | 38 | 10.82 |
|  | R Mid Orbital Gyrus (VMPFC) | 8 | 44 | -12 | 12 | 7.74 |
|  | L Mid Orbital Gyrus/ Area Fp2 (MPFC) | -2 | 58 | -6 | 26 | 7.54 |
|  | L Superior Medial Gyrus (MPFC) | 0 | 68 | 14 | 24 | 8.78 |
|  | R PCC | 0 | -50 | 20 | 36 | 12.39 |
|  | L Postcentral Gyrus/ Area 3b | -42 | -24 | 50 | 13 | 6.87 |

*^a^* This table presents brain regions that correlate with feedback-driven EV (derived from the across-subjects model fit to Uninstructed Group learners) within the entire Uninstructed Group (n = 38). Results are whole-brain FDR-corrected (q < .05) and clusters are defined based on contiguity with voxels at uncorrected p < .001 and p < .01.
