# Supplementary material for: Instructed knowledge shapes feedback-driven aversive learning in striatum and orbitofrontal cortex, but not the amygdala
Source: eLife. 2016 May 12;5:e15192. doi: 10.7554/eLife.15192 (PMC4907691; doi:10.7554/eLife.15192)
Supplement: Figure 3—figure supplement 2—source data 1. — This table presents brain regions that correlate with instruction-based EV (derived from the across-subjects model fit to Instructed Group learners) within Instructed Group learners (n = 20). Results are whole-brain FDR-corrected (q<0.05) and clusters are defined based on contiguity with voxels at uncorrected p<0.001 and p<0.01. DOI: http://dx.doi.org/10.7554/eLife.15192.012 [file elife-15192-fig3-figsupp2-data1.docx]

*Figure 3 – figure supplement 2 - Source data 1. Neural correlates of instruction-based EV: Instructed Group Learners (n = 20)^a^*

| **Contrast** | **Region** | **x** | **y** | **z** | **Number of voxels** | **Robust regression intercept** |
| --- | --- | --- | --- | --- | --- | --- |
| *Positive correlation with instruction-based EV* | R Cerebelum VIII | 14 | -54 | -58 | 23 | 9.51 |
|  | R Cerebelum VIII | 22 | -64 | -58 | 205 | 12.19 |
|  | Lobule VIIIa Hem | -22 | -68 | -56 | 33 | 10.62 |
|  | Bilateral Caudate, bilateral thalamus, midbrain, brainstem (contiguous) | 0 | -12 | -2 | 3147 | 24.37 |
|  | Lobule VIIIa Hem | -38 | -42 | -56 | 11 | 8.9 |
|  | L Cerebelum VIII | -24 | -54 | -52 | 34 | 8.74 |
|  | L Cerebelum VIII | -38 | -54 | -52 | 23 | 7.36 |
|  | Lobule VIIIa Verm | -4 | -76 | -48 | 17 | 7.2 |
|  | L Medial Temporal Pole | -32 | 12 | -44 | 12 | 7.57 |
|  | Lobule VIIIb Verm | 4 | -68 | -44 | 10 | 8.66 |
|  | R Inferior Temporal Gyrus | 38 | -2 | -42 | 32 | 10.32 |
|  | L Cerebelum Crus 1 | -36 | -50 | -32 | 87 | 13.17 |
|  | R Cerebelum Crus 1 | 42 | -46 | -34 | 12 | 7.29 |
|  | R Cerebelum IV-V | 16 | -48 | -20 | 258 | 11.96 |
|  | L Inferior Temporal Gyrus | -62 | -52 | -24 | 13 | 7.6 |
|  | L Insula Lobe | -30 | 8 | -16 | 65 | 11.2 |
|  | R Insula Lobe | 40 | 10 | 2 | 1080 | 19.04 |
|  | R IFG p. Orbitalis | 48 | 18 | -8 | 59 | 9.2 |
|  | L Insula, L Rolandic Operculum, SII (contiguous) | -46 | -6 | 12 | 2471 | 22.69 |
|  | Cerebellar Vermis 4/5 | 2 | -56 | -4 | 46 | 8.6 |
|  | L MCC | 0 | 12 | 40 | 2106 | 17 |
|  | R SupraMarginal Gyrus/ Area PFop (IPL) | 58 | -26 | 24 | 349 | 21.23 |
|  | L SupraMarginal Gyrus/ Area PFm (IPL) | -64 | -46 | 26 | 21 | 10.23 |
|  | L Middle Frontal Gyrus (latPFC) | -40 | 34 | 22 | 15 | 9.44 |
|  | R SupraMarginal Gyrus/ Area PFm (IPL) | 66 | -46 | 26 | 18 | 11.42 |
|  | L Middle Frontal Gyrus (DLPFC) | -40 | 46 | 30 | 213 | 12.47 |
|  | R Middle Frontal Gyrus (DLPFC) | 40 | 42 | 34 | 39 | 10.95 |
|  | R MCC | 12 | -22 | 40 | 89 | 8.57 |
|  | L MCC | -10 | -24 | 44 | 76 | 8.78 |
|  | RPrecentral Gyrus (DLPFC) | 50 | 6 | 46 | 199 | 15.72 |
|  | L Middle Frontal Gyrus (DLPFC) | -46 | 30 | 42 | 14 | 10.27 |
|  | L Precentral Gyrus | -40 | -2 | 52 | 38 | 7.54 |
|  | L Precentral Gyrus | -52 | -4 | 52 | 21 | 8.36 |
| *Negative correlation with instruction-based EV* | R Cerebelum Crus 2 | 26 | -88 | -42 | 14 | 13.09 |
|  | Cerebellar Vermis 9 | 0 | -48 | -42 | 13 | 9.34 |
|  | Lobule VIIa crusII Hem | -6 | -96 | -34 | 50 | 11 |
|  | L Cerebelum Crus 2 | -24 | -90 | -38 | 17 | 9.03 |
|  | L Medial Temporal Pole | -20 | 10 | -38 | 15 | 11.28 |
|  | Lobule VIIa crusII Hem | 10 | -94 | -30 | 38 | 7.53 |
|  | R Cerebelum Crus 2 | 20 | -90 | -32 | 20 | 7.98 |
|  | R Cerebelum Crus 1 | 34 | -82 | -28 | 24 | 7.64 |
|  | R Cerebelum Crus 1 | 50 | -74 | -26 | 10 | 8.82 |
|  | L Rectal Gyrus (VMPFC/mOFC) | -2 | 36 | -20 | 129 | 13.88 |
|  | R Middle Temporal Gyrus | 56 | -12 | -24 | 25 | 7.19 |
|  | R Fusiform Gyrus | 46 | -50 | -20 | 27 | 8.67 |
|  | R Inferior Temporal Gyrus | 60 | -60 | -20 | 10 | 8.6 |
|  | R Hippocampus (CA2) | 34 | -34 | -6 | 306 | 11.83 |
|  | L Hippocampus (CA3) | -22 | -16 | -16 | 20 | 10.06 |
|  | L IFG p. Orbitalis (latOFC) | -38 | 28 | -16 | 12 | 9.46 |
|  | R Middle Orbital Gyrus (latOFC) | 32 | 40 | -10 | 70 | 10.73 |
|  | L Mid Orbital Gyrus/ Area Fp2 (mOFC) | -6 | 54 | -10 | 94 | 9.96 |
|  | L Middle Orbital Gyrus (mOFC) | -26 | 42 | -6 | 43 | 8.49 |
|  | L Middle Orbital Gyrus (mOFC) | -40 | 44 | -4 | 213 | 12.48 |
|  | R Fusiform Gyrus/ Area hOc4v [V4(v)] | 28 | -74 | -4 | 43 | 7.82 |
|  | L Lingual Gyrus | -24 | -60 | -4 | 127 | 14.27 |
|  | L Mid Orbital Gyrus/ Area Fp2 (MPFC) | -2 | 64 | -2 | 25 | 12.84 |
|  | L Superior Medial Gyrus/ Area Fp1 (MPFC) | -8 | 62 | 12 | 581 | 18.87 |
|  | R Superior Temporal Gyrus/ Area TE 3 | 68 | -16 | 4 | 17 | 17.96 |
|  | L Middle Occipital Gyrus | -34 | -80 | 20 | 274 | 11.44 |
|  | R Middle Occipital Gyrus/ Area hOc4la | 44 | -78 | 14 | 132 | 14.67 |
|  | R Superior Medial Gyrus/ Area Fp1 | 14 | 60 | 12 | 34 | 8.12 |
|  | R Postcentral Gyrus/ Area 3b | 58 | -6 | 26 | 108 | 10.77 |
|  | L Angular Gyrus/ Area PGa (IPL) | -46 | -64 | 32 | 121 | 8.11 |
|  | R Superior Occipital Gyrus | 30 | -84 | 28 | 44 | 7.71 |
|  | R Angular Gyrus/ Area PGa (IPL) | 56 | -58 | 28 | 19 | 8.37 |
|  | R Precuneus | 4 | -60 | 34 | 79 | 11.3 |
|  | L Postcentral Gyrus | -64 | -10 | 34 | 26 | 8.72 |
|  | R Superior Medial Gyrus (DMPFC) | 14 | 54 | 30 | 10 | 10.05 |
|  | R Angular Gyrus/ Area PGp (IPL) | 46 | -64 | 38 | 36 | 8.33 |
|  | R MCC | 10 | -44 | 38 | 21 | 7.85 |
|  | R Superior Frontal Gyrus (DMPFC) | 16 | 28 | 38 | 10 | 8.55 |
|  | L Middle Frontal Gyrus (DLPFC) | -32 | 18 | 52 | 183 | 10.25 |
|  | L Superior Frontal Gyrus (DMPFC) | -12 | 38 | 46 | 115 | 9.41 |
|  | R Middle Frontal Gyrus (DMPFC) | 30 | 20 | 48 | 22 | 8.7 |
|  | R Superior Frontal Gyrus (DMPFC) | 16 | 38 | 48 | 34 | 7.29 |
|  | R Middle Frontal Gyrus (DMPFC) | 30 | 22 | 56 | 23 | 7.49 |

*^a^ Figure 3 – figure supplement 2 - Source data 1. Neural correlates of instruction-based EV: Instructed Group Learners (n = 20)*. This table presents brain regions that correlate with instruction-based EV (derived from the across-subjects model fit to Instructed Group learners) within Instructed Group learners (n = 20). Results are whole-brain FDR-corrected (q < .05) and clusters are defined based on contiguity with voxels at uncorrected p < .001 and p < .01.
