# Supplementary material for: Instructed knowledge shapes feedback-driven aversive learning in striatum and orbitofrontal cortex, but not the amygdala
Source: eLife. 2016 May 12;5:e15192. doi: 10.7554/eLife.15192 (PMC4907691; doi:10.7554/eLife.15192)
Supplement: Figure 3—figure supplement 2—source data 2. — This table presents brain regions that correlate with instruction-based EV (derived from the across-subjects model fit to Instructed Group learners) within the entire Instructed Group (n = 30). Results are whole-brain FDR-corrected (q<0.05) and clusters are defined based on contiguity with voxels at uncorrected p<0.001 and p<0.01. DOI: http://dx.doi.org/10.7554/eLife.15192.013 [file elife-15192-fig3-figsupp2-data2.docx]

*Figure 3 – figure supplement 2 - Source data 2. Neural correlates of instruction-based EV: Entire Instructed Group (n = 30)^a^*

| **Contrast** | **Region** | **x** | **y** | **z** | **Number of voxels** | **Robust regression intercept** |
| --- | --- | --- | --- | --- | --- | --- |
| *Positive correlation with instruction-based EV* | R Cerebelum VIII | 20 | -62 | -58 | 357 | 13.37 |
|  | L Cerebelum VIII | -38 | -56 | -56 | 87 | 9.58 |
|  | Lobule VIIIa Hem | -22 | -70 | -56 | 17 | 7.82 |
|  | L Cerebelum VIII | -22 | -54 | -52 | 15 | 6.36 |
|  | Bilateral caudate, bilateral thalamus, midbrain, brainstem (contiguous) | 0 | -12 | 2 | 3372 | 30.97 |
|  | R Inferior Temporal Gyrus | 40 | -4 | -42 | 39 | 12.83 |
|  | L Cerebelum VII | -30 | -36 | -40 | 31 | 7.55 |
|  | L Medial Temporal Pole | -32 | 12 | -42 | 12 | 6.83 |
|  | R Cerebelum IV-V | 10 | -54 | -16 | 432 | 16.9 |
|  | L Cerebelum Crus 1 | -36 | -52 | -32 | 94 | 8.75 |
|  | L Pons | -8 | -30 | -34 | 12 | 6.97 |
|  | R Cerebelum VI | 34 | -54 | -30 | 14 | 6.21 |
|  | L Inferior Temporal Gyrus | -40 | -24 | -26 | 31 | 6.8 |
|  | R Fusiform Gyrus | 40 | -20 | -28 | 13 | 6.41 |
|  | L Inferior Temporal Gyrus | -44 | -40 | -24 | 49 | 9.32 |
|  | R Insula Lobe | 40 | 10 | 4 | 1243 | 17.28 |
|  | Cerebellar Vermis 3 | 0 | -46 | -20 | 26 | 8.67 |
|  | R Inferior Temporal Gyrus | 60 | -40 | -20 | 10 | 6.47 |
|  | L Insula, L Rolandic Operculum/ Area OP4 [PV], SII (Contiguous) | -48 | -10 | 12 | 3682 | 22.63 |
|  | R Middle Temporal Gyrus | 52 | -22 | -10 | 41 | 12.22 |
|  | R IFG p. Orbitalis | 52 | 20 | -10 | 41 | 7.94 |
|  | L Middle Temporal Gyrus | -52 | -56 | 10 | 171 | 9.1 |
|  | R IFG p. Opercularis/ Area 45 | 54 | 18 | 8 | 15 | 7.01 |
|  | R SupraMarginal Gyrus/ Area PFcm (IPL) | 58 | -28 | 22 | 1028 | 22.14 |
|  | R Superior Temporal Gyrus | 46 | -40 | 14 | 60 | 8.38 |
|  | L MCC | 0 | 6 | 40 | 3553 | 20.85 |
|  | R IFG p. Triangularis/ Area 45 | 56 | 20 | 18 | 12 | 6.59 |
|  | L Middle Frontal Gyrus | -36 | 36 | 22 | 42 | 9.08 |
|  | L Middle Frontal Gyrus | -40 | 46 | 32 | 258 | 13.23 |
|  | R SupraMarginal Gyrus/ Area PFm (IPL) | 52 | -44 | 36 | 14 | 8.1 |
|  | R Middle Frontal Gyrus (DLPFC) | 40 | 42 | 34 | 62 | 11.1 |
|  | RPrecentral Gyrus (DLPFC) | 50 | 4 | 48 | 391 | 23.82 |
|  | L Middle Frontal Gyrus (DLPFC) | -46 | 30 | 42 | 14 | 9.32 |
|  | R Middle Frontal Gyrus (DLPFC) | 38 | 38 | 44 | 20 | 7.43 |
|  | L Precentral Gyrus | -42 | -6 | 54 | 235 | 12.94 |
|  | L Postcentral Gyrus/ Area 4p | -32 | -26 | 52 | 37 | 15.32 |
| *Negative correlation with instruction-based EV* | Lobule IX Hem | 0 | -46 | -48 | 41 | 6.74 |
|  | R Cerebelum Crus 2 | 26 | -86 | -42 | 12 | 6.91 |
|  | L Medial Temporal Pole | -20 | 12 | -38 | 14 | 9.22 |
|  | R Cerebelum Crus 1 | 34 | -84 | -30 | 91 | 8.78 |
|  | R Cerebelum Crus 2 | 22 | -92 | -32 | 55 | 11.03 |
|  | R Inferior Temporal Gyrus | 52 | -6 | -32 | 10 | 6.32 |
|  | R Middle Temporal Gyrus | 46 | 4 | -30 | 24 | 7.25 |
|  | L Middle Temporal Gyrus | -42 | 4 | -30 | 10 | 8.61 |
|  | R Cerebelum Crus 1 | 48 | -66 | -26 | 105 | 12.34 |
|  | R Cerebelum Crus 1 | 36 | -70 | -28 | 10 | 9.82 |
|  | L Cerebelum Crus 1 | -36 | -86 | -26 | 13 | 6.64 |
|  | R Middle Temporal Gyrus | 56 | -12 | -24 | 45 | 10.36 |
|  | R Hippocampus (CA2) | 36 | -30 | -8 | 522 | 14.55 |
|  | L Amygdala (LB)/ Hippocampus | -30 | -6 | -22 | 23 | 7.77 |
|  | L Superior Medial Gyrus/ Area Fp2 | -6 | 56 | 2 | 1477 | 15.17 |
|  | L Cerebelum IV-V | -14 | -54 | -20 | 13 | 8.13 |
|  | L Middle Temporal Gyrus | -60 | -12 | -22 | 12 | 6.7 |
|  | R ParaHippocampal Gyrus/ Subiculum | 20 | -26 | -18 | 32 | 8.86 |
|  | L Hippocampus (CA2) | -38 | -28 | -12 | 192 | 12.78 |
|  | L Middle Temporal Gyrus | -56 | -26 | -16 | 27 | 9.83 |
|  | L Hippocampus (CA3) | -22 | -16 | -16 | 45 | 12.16 |
|  | R Hippocampus/ HATA Region | 20 | -10 | -18 | 13 | 6.27 |
|  | L IFG p. Orbitalis (latOFC) | -36 | 30 | -16 | 13 | 6.9 |
|  | R pgACC, Caudate, MPFC (contiguous) | 22 | 34 | 12 | 790 | 12.72 |
|  | L Middle Orbital Gyrus (latPFC) | -38 | 46 | -6 | 112 | 7.66 |
|  | R Fusiform Gyrus/ Area FG1 | 34 | -68 | -10 | 14 | 11.96 |
|  | R Lingual Gyrus/ Area hOc3v [V3v] | 20 | -70 | -6 | 61 | 8.88 |
|  | L Inferior Occipital Gyrus | -34 | -64 | -2 | 124 | 14.29 |
|  | L Calcarine Gyrus | 4 | -98 | 12 | 158 | 12.76 |
|  | L Inferior Occipital Gyrus/ Area hOc4lp | -28 | -88 | -4 | 16 | 7.91 |
|  | R Fusiform Gyrus | 28 | -74 | -2 | 32 | 7.27 |
|  | L Lingual Gyrus/ Area hOc2 [V2] | -8 | -68 | 0 | 32 | 7.29 |
|  | R Lingual Gyrus | 28 | -58 | -4 | 12 | 6.21 |
|  | L IFG p. Triangularis (latPFC) | -44 | 38 | -2 | 19 | 6.22 |
|  | L Lingual Gyrus/ Area hOc3v [V3v] | -20 | -68 | -2 | 11 | 11.76 |
|  | R Middle Occipital Gyrus/ Area hOc4la | 44 | -78 | 14 | 242 | 19.06 |
|  | L Middle Occipital Gyrus | -32 | -80 | 20 | 453 | 11.96 |
|  | L IFG p. Triangularis/ BA45 (latPFC) | -50 | 26 | 8 | 12 | 6.45 |
|  | R Middle Occipital Gyrus | 30 | -72 | 22 | 12 | 6.79 |
|  | R Superior Occipital Gyrus | 28 | -84 | 26 | 61 | 10.07 |
|  | R Postcentral Gyrus | 62 | -8 | 30 | 36 | 7.73 |
|  | L Precuneus | 2 | -60 | 32 | 108 | 9.23 |
|  | L Angular Gyrus/ Area PGa (IPL) | -46 | -64 | 30 | 106 | 8.28 |
|  | R Postcentral Gyrus/ Area 4p | 50 | -6 | 28 | 11 | 9.46 |
|  | L Postcentral Gyrus/ Area 3a | -44 | -16 | 30 | 14 | 7.6 |
|  | R Superior Occipital Gyrus | 28 | -68 | 34 | 33 | 7.75 |
|  | R Superior Medial Gyrus (DMPFC) | 14 | 52 | 30 | 12 | 8.93 |
|  | L Middle Frontal Gyrus (DMPFC) | -32 | 16 | 48 | 68 | 7.53 |
|  | L Superior Frontal Gyrus (DMPFC) | -14 | 34 | 46 | 104 | 12.51 |
|  | R Middle Frontal Gyrus (DMPFC) | 28 | 20 | 48 | 36 | 10.64 |
|  | RPrecentral Gyrus | 34 | -24 | 54 | 45 | 7.59 |
|  | R Postcentral Gyrus | 46 | -22 | 54 | 15 | 8.85 |
|  | L Middle Frontal Gyrus (DMPFC) | -32 | 12 | 60 | 67 | 9.99 |
|  | L Posterior-Medial Frontal (DMPFC) | -2 | 18 | 66 | 18 | 6.49 |

*^a^* This table presents brain regions that correlate with instruction-based EV (derived from the across-subjects model fit to Instructed Group learners) within the entire Instructed Group (n = 30). Results are whole-brain FDR-corrected (q < .05) and clusters are defined based on contiguity with voxels at uncorrected p < .001 and p < .01.
