# Supplementary material for: Instructed knowledge shapes feedback-driven aversive learning in striatum and orbitofrontal cortex, but not the amygdala
Source: eLife. 2016 May 12;5:e15192. doi: 10.7554/eLife.15192 (PMC4907691; doi:10.7554/eLife.15192)
Supplement: Figure 4—figure supplement 1—source data 1. — This table presents brain regions that show preferential correlations with either feedback-driven or instruction-based EV, based on direct contrasts between the two signals. Analyses are restricted to Instructed Group learners (n = 20). Results are whole-brain FDR-corrected (q < 0.05) and clusters are defined based on contiguity with voxels at uncorrected p<0.001 and p<0.01. DOI: http://dx.doi.org/10.7554/eLife.15192.017 [file elife-15192-fig4-figsupp1-data1.docx]

*Figure 4 – figure supplement 1 - Source data 1. Feedback-driven vs instruction-based EV: Instructed Group Learners (n = 20)*^a^

| **Contrast** | **Region** | **x** | **y** | **z** | **Number of voxels** | **Robust regression intercept** |
| --- | --- | --- | --- | --- | --- | --- |
| *Feedback-driven > instruction-based (positive)* | L Inferior Temporal Gyrus | -38 | 8 | -42 | 129 | 9.74 |
|  | Lobule VIIa crusII Hem | -4 | -86 | -50 | 13 | 8.84 |
|  | R Cerebelum IX | 2 | -56 | -50 | 16 | 9.71 |
|  | L Cerebelum Crus 2 | -56 | -52 | -46 | 12 | 11.18 |
|  | R Cerebelum VIII | 26 | -70 | -44 | 15 | 11.63 |
|  | R Medial Temporal Pole | 32 | 8 | -42 | 46 | 8.58 |
|  | R Fusiform Gyrus | 38 | -10 | -38 | 113 | 15.6 |
|  | L Fusiform Gyrus | -24 | -6 | -40 | 41 | 9.32 |
|  | L Medial Temporal Pole | -34 | 24 | -38 | 57 | 11.56 |
|  | R Inferior Temporal Gyrus | 54 | -6 | -32 | 25 | 14.66 |
|  | R Temporal Pole | 32 | 10 | -28 | 81 | 13.19 |
|  | L Fusiform Gyrus | -34 | -14 | -28 | 16 | 14.51 |
|  | R Medial Temporal Pole | 54 | 10 | -28 | 20 | 9.09 |
|  | R Cerebelum Crus 1 | 12 | -78 | -26 | 23 | 9.44 |
|  | R Fusiform Gyrus | 40 | -18 | -22 | 29 | 8.83 |
|  | R Lingual Gyrus/ Area hOc1 [V1] | 12 | -78 | 0 | 5932 | 16.83 |
|  | R Fusiform Gyrus | 28 | -46 | -10 | 261 | 13.1 |
|  | R Amygdala (SF), ParaHippocampal Gyrus (contiguous) | 18 | -6 | -20 | 173 | 17.83 |
|  | R Middle Temporal Gyrus | 66 | -6 | -22 | 51 | 24.54 |
|  | L IFG p. Orbitalis | -32 | 28 | -20 | 31 | 9.01 |
|  | R Inferior Temporal Gyrus | 48 | -54 | -18 | 19 | 10.87 |
|  | L Amygdala, Hippocampus (contiguous) | -18 | -12 | -18 | 125 | 12.83 |
|  | R Fusiform Gyrus | 42 | -30 | -16 | 12 | 12.26 |
|  | L IFG p. Orbitalis (latOFC) | -52 | 34 | -14 | 21 | 13.59 |
|  | L Middle Temporal Gyrus | -60 | -30 | -4 | 355 | 16.5 |
|  | L IFG p. Triangularis (latPFC) | -46 | 42 | -2 | 208 | 10.7 |
|  | R Middle Temporal Gyrus/ Area TE 3 | 66 | -26 | -2 | 97 | 10.36 |
|  | R Thalamus | 22 | -26 | 6 | 17 | 11.67 |
|  | L Superior Medial Gyrus/ Area Fp2 (MPFC) | -2 | 60 | 10 | 106 | 10.16 |
|  | L Middle Frontal Gyrus (DLPFC) | -40 | 14 | 40 | 1004 | 15.96 |
|  | R IFG p. Triangularis (DLPFC) | 52 | 32 | 24 | 193 | 11.81 |
|  | RPrecentral Gyrus | 54 | 0 | 26 | 504 | 16.83 |
|  | L Angular Gyrus/ Area PFm (IPL) | -46 | -58 | 24 | 163 | 10.92 |
|  | R Inferior Parietal Lobule / Area PFm (IPL) | 60 | -50 | 38 | 16 | 10.11 |
|  | L Inferior Parietal Lobule/ Area hIP2 (IPS) | -42 | -46 | 42 | 31 | 11 |
|  | R SupraMarginal Gyrus/ Area hIP2 (IPS) | 50 | -38 | 44 | 37 | 8.62 |
|  | RPrecentral Gyrus | 50 | -16 | 50 | 77 | 10.55 |
|  | R Middle Frontal Gyrus (DLPFC) | 32 | 22 | 56 | 603 | 13.17 |
|  | L Precentral Gyrus (DLPFC) | -48 | 4 | 54 | 17 | 10.77 |
|  | L Superior Medial Gyrus (DMPFC) | -2 | 44 | 54 | 13 | 9.92 |
| *Instruction-based > Feedback-driven (negative)* | R Cerebelum VIII | 36 | -56 | -62 | 20 | 8.56 |
|  | R Cerebelum VIII | 16 | -66 | -56 | 19 | 9.59 |
|  | R Cerebelum Crus 2 | 52 | -50 | -50 | 14 | 8.82 |
|  | L Cerebelum VIII | -14 | -62 | -42 | 13 | 12.61 |
|  | Pons | 0 | -34 | -42 | 18 | 8.91 |
|  | L Cerebelum VI | -34 | -50 | -34 | 30 | 12.07 |
|  | L Inferior Temporal Gyrus | -54 | -14 | -36 | 14 | 9.54 |
|  | Lobule VIIa crusI Hem | -32 | -90 | -30 | 13 | 11.27 |
|  | R Cerebelum VI | 22 | -52 | -22 | 110 | 11.89 |
|  | Midbrain including PAG | 2 | -20 | -10 | 305 | 11.46 |
|  | R Insula Lobe | 34 | 20 | 4 | 221 | 12.95 |
|  | Area Id1 | 40 | -16 | -6 | 15 | 14.01 |
|  | R ACC | 2 | 42 | 0 | 23 | 10.62 |
|  | R Pallidum/ Putamen | 20 | 0 | -2 | 16 | 8.75 |
|  | Cerebellar Vermis 4/5 | 0 | -52 | 2 | 11 | 9.02 |
|  | L Rolandic Operculum | -48 | 2 | 6 | 203 | 9.66 |
|  | L Caudate Nucleus | -8 | 12 | 2 | 14 | 14.59 |
|  | L Thalamus | -10 | -18 | 6 | 67 | 8.59 |
|  | Thal: Prefrontal | -14 | -4 | 10 | 38 | 14.87 |
|  | L ACC | 2 | 36 | 22 | 226 | 12.28 |
|  | L Rolandic Operculum | -44 | -22 | 18 | 32 | 8.36 |

^a^ This table presents brain regions that show preferential correlations with either feedback-driven or instruction-based EV, based on direct contrasts between the two signals. Analyses are restricted to Instructed Group learners (n = 20). Results are whole-brain FDR-corrected (q < .05) and clusters are defined based on contiguity with voxels at uncorrected p < .001 and p < .01.
