# Supplementary material for: Instructed knowledge shapes feedback-driven aversive learning in striatum and orbitofrontal cortex, but not the amygdala
Source: eLife. 2016 May 12;5:e15192. doi: 10.7554/eLife.15192 (PMC4907691; doi:10.7554/eLife.15192)
Supplement: Figure 4—figure supplement 1—source data 2. — This table presents brain regions that show preferential correlations with either feedback-driven or instruction-based EV, based on direct contrasts between the two signals. Analyses include the entire Instructed Group (n = 30). Results are whole-brain FDR-corrected (q < 0.05) and clusters are defined based on contiguity with voxels at uncorrected p<0.001 and p<0.01. DOI: http://dx.doi.org/10.7554/eLife.15192.018 [file elife-15192-fig4-figsupp1-data2.docx]

*Figure 4 – figure supplement 1 - Source data 2. Feedback-driven vs instruction-based EV: Entire Instructed Group (n = 30)*^a^

| **Contrast** | **Region** | **x** | **y** | **z** | **Number of voxels** | **Robust regression intercept** |
| --- | --- | --- | --- | --- | --- | --- |
| *Feedback-driven > instruction-based (positive)* | R Cerebelum IX | 2 | -56 | -50 | 33 | 10.56 |
|  | L Cerebelum Crus 2 | -54 | -52 | -48 | 11 | 7.33 |
|  | R Fusiform Gyrus | 38 | -6 | -38 | 390 | 12.55 |
|  | R Cerebelum Crus 2 | 46 | -54 | -44 | 19 | 9.53 |
|  | R Cerebelum IX | 2 | -46 | -42 | 15 | 8.61 |
|  | L Inferior Temporal Gyrus | -48 | 6 | -38 | 122 | 10.53 |
|  | L Fusiform Gyrus | -26 | -6 | -36 | 38 | 7.41 |
|  | L Medial Temporal Pole | -18 | 6 | -36 | 25 | 8.13 |
|  | L Rectal Gyrus (mOFC/VMPFC) | -2 | 34 | -24 | 314 | 10.16 |
|  | L Amygdala, Hippocampus (contiguous) | -20 | -12 | -18 | 187 | 15 |
|  | R Temporal Pole/ Parahippocampal gyrus | 32 | 10 | -28 | 105 | 12.32 |
|  | L Temporal Pole | -32 | 8 | -30 | 11 | 9.57 |
|  | L Fusiform Gyrus | -36 | -14 | -28 | 17 | 8.64 |
|  | R Lingual Gyrus/ Area hOc1 [V1] | 6 | -70 | 2 | 8942 | 19.54 |
|  | L Inferior Temporal Gyrus | -60 | -60 | -22 | 14 | 11.87 |
|  | R Amygdala (LB), Hippocampus (contigous) | 20 | -10 | -20 | 263 | 15.42 |
|  | R Middle Temporal Gyrus | 64 | -2 | -22 | 69 | 11.24 |
|  | R IFG p. Orbitalis | 26 | 28 | -20 | 18 | 7.17 |
|  | R Middle Temporal Gyrus | 64 | -14 | -20 | 37 | 7.95 |
|  | L IFG p. Orbitalis | -34 | 30 | -18 | 40 | 6.56 |
|  | L Cerebelum Crus 1 | -22 | -88 | -22 | 15 | 10.44 |
|  | R Middle Orbital Gyrus | 44 | 46 | -18 | 18 | 7.01 |
|  | L Fusiform Gyrus | -26 | -36 | -16 | 34 | 9.11 |
|  | R Middle Temporal Gyrus | 50 | -8 | -18 | 18 | 7.42 |
|  | R Temporal Pole | 60 | 12 | -16 | 10 | 9.03 |
|  | L Middle Temporal Gyrus | -48 | -18 | -14 | 11 | 7.17 |
|  | L Caudate Nucleus/ BA33 | -4 | 8 | -12 | 18 | 7.19 |
|  | L IFG p. Orbitalis | -52 | 34 | -14 | 23 | 8.72 |
|  | L Middle Temporal Gyrus | -58 | -38 | -4 | 408 | 13.38 |
|  | L Middle Temporal Gyrus | -64 | -22 | -10 | 56 | 9.53 |
|  | L Middle Temporal Gyrus | -62 | -12 | -12 | 39 | 10.08 |
|  | L IFG p. Triangularis | -46 | 42 | -2 | 173 | 9.9 |
|  | L Superior Frontal Gyrus | -20 | 50 | 2 | 36 | 9.34 |
|  | L IFG p. Opercularis (DLPFC) | -46 | 14 | 34 | 1683 | 12.6 |
|  | L Calcarine Gyrus/ Area hOc1 [V1] | -18 | -78 | 6 | 17 | 8.59 |
|  | L Superior Medial Gyrus/ Area Fp2 | -4 | 62 | 10 | 166 | 11.16 |
|  | R Middle Frontal Gyrus (DLPFC) | 40 | 26 | 44 | 1042 | 14.39 |
|  | R Postcentral Gyrus | 64 | -4 | 24 | 270 | 19.88 |
|  | L Angular Gyrus | -46 | -56 | 26 | 186 | 10.02 |
|  | R IFG p. Opercularis | 46 | 8 | 28 | 226 | 8.89 |
|  | L Superior Frontal Gyrus (DMPFC) | -16 | 46 | 44 | 344 | 11.89 |
|  | R Precuneus | 4 | -58 | 40 | 72 | 8.62 |
|  | R Superior Frontal Gyrus (DMPFC) | 22 | 36 | 40 | 43 | 9.39 |
|  | L MCC | -4 | -38 | 40 | 25 | 8.17 |
|  | L Inferior Parietal Lobule/ Area hIP1 (IPS) | -40 | -40 | 40 | 21 | 7.29 |
|  | Area 3a | 34 | -18 | 38 | 10 | 7.33 |
|  | L Superior Medial Gyrus (DMPFC) | -6 | 28 | 44 | 21 | 6.83 |
|  | R Superior Frontal Gyrus (DMPFC) | 16 | 44 | 46 | 98 | 8.61 |
|  | R Inferior Parietal Lobule/ Area hIP2 (IPS) | 50 | -42 | 46 | 54 | 7.4 |
|  | R Postcentral Gyrus/ BA1 | 54 | -20 | 50 | 120 | 21.1 |
|  | L Inferior Parietal Lobule / Area PF (IPL) | -52 | -40 | 50 | 33 | 9.73 |
|  | L Middle Frontal Gyrus (DMPFC) | -22 | 6 | 52 | 53 | 14.15 |
| *Instruction-based > Feedback-driven (negative)* | R Cerebelum VIII | 36 | -58 | -62 | 26 | 7.03 |
|  | R Cerebelum VIII | 16 | -68 | -56 | 119 | 11.52 |
|  | L Cerebelum VIII | -28 | -56 | -60 | 11 | 7.79 |
|  | R Cerebelum VIII | 22 | -54 | -52 | 15 | 8.44 |
|  | Cerebellar Vermis 8 | 4 | -68 | -40 | 50 | 10.57 |
|  | Bilateral NAcc,Bilateral Caudate, Bilateral Putamen, Bilateral Thalamus, Midbrain, Brainstem (contiguous) | -4 | -12 | -6 | 1744 | 17.21 |
|  | L Cerebelum VI | -34 | -52 | -32 | 104 | 11.62 |
|  | R Cerebelum IV-V | 20 | -52 | -20 | 278 | 17.54 |
|  | L Cerebelum VI | -24 | -62 | -24 | 38 | 9.52 |
|  | R Insula Lobe | 36 | 20 | 0 | 702 | 14.46 |
|  | R Hippocampus | 28 | -12 | -10 | 21 | 7.52 |
|  | L Hippocampus | -28 | -12 | -10 | 23 | 7.64 |
|  | Area Id1 | 40 | -16 | -6 | 56 | 20.35 |
|  | L Insula Lobe | -40 | 10 | 4 | 774 | 19.83 |
|  | Area Ig2 | -38 | -22 | -2 | 25 | 6.95 |
|  | R Rolandic Operculum | 54 | 2 | 4 | 103 | 9.83 |
|  | L Putamen | -20 | 8 | -2 | 13 | 8.22 |
|  | R rACC | 6 | 42 | 8 | 12 | 6.94 |
|  | L rACC | -8 | 34 | 14 | 67 | 11.12 |
|  | L SupraMarginal Gyrus/ Area OP1 [SII] | -52 | -28 | 22 | 672 | 11.57 |
|  | L MCC | -2 | -8 | 40 | 2467 | 19.83 |
|  | R SupraMarginal Gyrus/ Area PFcm (IPL) | 54 | -30 | 24 | 191 | 15.06 |
|  | R Precuneus | 14 | -62 | 32 | 26 | 6.9 |
|  | L Postcentral Gyrus/ BA 3b | -38 | -26 | 46 | 89 | 14.26 |
|  | L Postcentral Gyrus | -44 | -12 | 48 | 17 | 8.21 |
|  | L Precentral Gyrus | -26 | -12 | 54 | 41 | 9.6 |

^a^ This table presents brain regions that show preferential correlations with either feedback-driven or instruction-based EV, based on direct contrasts between the two signals. Analyses include the entire Instructed Group (n = 30). Results are whole-brain FDR-corrected (q < .05) and clusters are defined based on contiguity with voxels at uncorrected p < .001 and p < .01.
