# Supplementary material for: Instructed knowledge shapes feedback-driven aversive learning in striatum and orbitofrontal cortex, but not the amygdala
Source: eLife. 2016 May 12;5:e15192. doi: 10.7554/eLife.15192 (PMC4907691; doi:10.7554/eLife.15192)
Supplement: Figure 5—figure supplement 1—source data 1. — This table presents brain regions whose differential responses (CS+ vs CS-) reversed immediately upon instruction. Regions that were positive in this contrast show greater activation with the current CS+ relative to the current CS-, whereas those that are negative show deactivation to the CS+ or increases with the CS-. Analyses are restricted to Instructed Group learners (n = 20). Results are whole-brain FDR-corrected (q<0.05) and clusters are defined based on contiguity with voxels at uncorrected p<0.001 and p<0.01. DOI: http://dx.doi.org/10.7554/eLife.15192.021 [file elife-15192-fig5-figsupp1-data1.docx]

*Figure 5 – figure supplement 1 – Source data 1. Immediate reversal with instructions (CS x Phase interaction): Instructed Group Learners (n = 20)*^a^

| **Contrast** | **Region** | **x** | **y** | **z** | **Number of voxels** | **Robust regression intercept** |
| --- | --- | --- | --- | --- | --- | --- |
| *Positive* | R Cerebelum VIII | 26 | -58 | -58 | 30 | 9.43 |
|  | R Cerebelum VI | 40 | -42 | -32 | 13 | 9.2 |
|  | L NAcc, L Thalamus, Midbrain (including PAG); contiguous | -4 | -20 | 0 | 379 | 12.82 |
|  | Ventral Thalamic Nucleus | 16 | -16 | -8 | 14 | 11.05 |
|  | R Pallidum | 12 | 6 | -4 | 72 | 11.33 |
|  | R Insula Lobe | 36 | 20 | 2 | 349 | 15.63 |
|  | L Insula Lobe | -36 | 22 | 0 | 39 | 9.06 |
|  | L Rolandic Operculum | -50 | -2 | 6 | 76 | 11.66 |
|  | L ACC | 2 | 38 | 20 | 185 | 14.67 |
|  | L SupraMarginal Gyrus/ Area PFop (IPL) | -60 | -30 | 26 | 188 | 18.24 |
|  | L MCC | -2 | 8 | 42 | 486 | 14.24 |
|  | R MCC | 6 | -18 | 34 | 15 | 9.54 |
|  | RPrecentral Gyrus | 46 | -4 | 40 | 22 | 10.48 |
|  | L Precentral Gyrus | -46 | -10 | 60 | 18 | 9.95 |
| *Negative* | R Cerebelum Crus 2 | 28 | -80 | -40 | 62 | 9.38 |
|  | L Cerebelum VIII | -26 | -64 | -42 | 11 | 9.22 |
|  | R IFG p. Orbitalis (latOFC) | 36 | 30 | -22 | 148 | 10.33 |
|  | L Medial Temporal Pole | -24 | 14 | -38 | 66 | 12.06 |
|  | L Medial Temporal Pole | -42 | 12 | -36 | 21 | 9.72 |
|  | R ParaHippocampal Gyrus/ Subiculum | 22 | -20 | -22 | 90 | 11.73 |
|  | L Hippocampus (CA3) | -26 | -18 | -20 | 52 | 11.06 |
|  | R ParaHippocampal Gyrus / Hippocampus (CA1) | 34 | -12 | -28 | 10 | 11.93 |
|  | R Middle Temporal Gyrus | 62 | -8 | -18 | 100 | 13.23 |
|  | L Rectal Gyrus (mOFC / VMPFC) | -2 | 34 | -20 | 298 | 10.16 |
|  | R Lingual Gyrus | 30 | -46 | -8 | 587 | 17.2 |
|  | L Inferior Temporal Gyrus | -38 | -30 | -16 | 82 | 10.13 |
|  | L Olfactory cortex/ Area 33 | -4 | 8 | -16 | 51 | 9.45 |
|  | R Cerebelum VI/ Area hOc2 [V2] | 16 | -86 | -16 | 60 | 13.56 |
|  | R Lingual Gyrus/ Area hOc1 [V1] | 18 | -72 | 0 | 3228 | 16.63 |
|  | L Middle Occipital Gyrus/ Area hOc5 [V5/MT] | -40 | -74 | 12 | 1390 | 15.81 |
|  | L Middle Orbital Gyrus | -42 | 46 | -2 | 180 | 12.86 |
|  | R Inferior Occipital Gyrus/ Area hOc4la | 46 | -76 | -10 | 22 | 9.07 |
|  | L Middle Temporal Gyrus/ Area TE 3 | -62 | -10 | -6 | 61 | 14.26 |
|  | R Middle Occipital Gyrus | 28 | -88 | 0 | 13 | 8.98 |
|  | L Superior Medial Gyrus/ Area Fp2 (MPFC) | -2 | 62 | 8 | 314 | 14.46 |
|  | R Putamen | 30 | -8 | 6 | 44 | 10.68 |
|  | L Middle Frontal Gyrus | -28 | 50 | 6 | 67 | 9.95 |
|  | L Insula Lobe | -36 | -8 | 10 | 48 | 11.63 |
|  | L Superior Temporal Gyrus/ Area TE 3 | -66 | -12 | 6 | 11 | 9.33 |
|  | R Postcentral Gyrus/ Area 3b | 58 | -6 | 24 | 618 | 16.34 |
|  | L IFG p. Triangularis/ Area 45 | -52 | 26 | 12 | 87 | 9.91 |
|  | L Precuneus | -6 | -58 | 12 | 37 | 12.2 |
|  | R Precuneus | 20 | -58 | 14 | 102 | 9.84 |
|  | L Postcentral Gyrus/ Area 3b | -58 | -10 | 30 | 320 | 12.89 |
|  | R Cuneus/ Area hOc2 [V2] | 8 | -86 | 14 | 12 | 11.77 |
|  | R Heschls Gyrus/ Area TE 1.1 | 38 | -28 | 16 | 19 | 9.22 |
|  | R Caudate Nucleus | 20 | -6 | 22 | 19 | 10.36 |
|  | L Precuneus | 0 | -64 | 28 | 222 | 12.97 |
|  | L IFG p. Opercularis (DLPFC) | -42 | 14 | 34 | 177 | 12.71 |
|  | R IFG p. Opercularis (DLPFC) | 42 | 10 | 24 | 20 | 9.46 |
|  | L Inferior Parietal Lobule | -36 | -38 | 38 | 51 | 13.92 |
|  | L Middle Frontal Gyrus (DLPFC) | -28 | 22 | 52 | 479 | 13.89 |
|  | R SupraMarginal Gyrus/ Area PFt (IPL) | 60 | -26 | 44 | 24 | 9.19 |
|  | RPrecentral Gyrus | 48 | -16 | 52 | 153 | 13.27 |
|  | R Middle Frontal Gyrus (DMPFC) | 32 | 22 | 56 | 46 | 9.12 |

^a^ This table presents brain regions whose differential responses (CS+ vs CS-) reversed immediately upon instruction. Regions that were positive in this contrast show greater activation with the current CS+ relative to the current CS-, whereas those that are negative show deactivation to the CS+ or increases with the CS-. Analyses are restricted to Instructed Group learners (n = 20). Results are whole-brain FDR-corrected (q < .05) and clusters are defined based on contiguity with voxels at uncorrected p < .001 and p < .01.
