# Supplementary material for: Instructed knowledge shapes feedback-driven aversive learning in striatum and orbitofrontal cortex, but not the amygdala
Source: eLife. 2016 May 12;5:e15192. doi: 10.7554/eLife.15192 (PMC4907691; doi:10.7554/eLife.15192)
Supplement: Figure 5—figure supplement 1—source data 2. — This table presents brain regions whose differential responses (CS+ vs CS-) reversed immediately upon instruction. Analyses include the entire Instructed Group (n = 30). Results are whole-brain FDR-corrected (q<0.05) and clusters are defined based on contiguity with voxels at uncorrected p<0.001 and p<0.01. DOI: http://dx.doi.org/10.7554/eLife.15192.022 [file elife-15192-fig5-figsupp1-data2.docx]

*Figure 5 – figure supplement 1 – Source data 2. Immediate reversal with instructions (CS x Phase interaction): Entire Instructed Group (n = 30)*^a^

| **Contrast** | **Region** | **x** | **y** | **z** | **Number of voxels** | **Robust regression intercept** |
| --- | --- | --- | --- | --- | --- | --- |
| *Positive* | R Cerebelum VIII | 26 | -60 | -58 | 43 | 10.63 |
|  | R Cerebelum VIII | 16 | -60 | -54 | 71 | 8.31 |
|  | L Cerebelum VIII | -38 | -60 | -50 | 12 | 9.71 |
|  | R Cerebelum VIII | 14 | -74 | -48 | 20 | 8.48 |
|  | L Cerebelum Crus 2 | -28 | -76 | -46 | 14 | 10.88 |
|  | R Cerebelum VI | 42 | -40 | -30 | 30 | 9.63 |
|  | L Cerebelum VI | -36 | -54 | -28 | 31 | 11.31 |
|  | R Cerebelum IV-V | 22 | -46 | -24 | 87 | 9.69 |
|  | R Inferior Temporal Gyrus | 64 | -38 | -28 | 10 | 16.08 |
|  | Bilateral caudate, bilateral thalamus, midbrain, brainstem (continuous) | 2 | -14 | 0 | 1325 | 16.75 |
|  | R Insula Lobe | 36 | 20 | 0 | 664 | 15.7 |
|  | R Insula Lobe | 40 | -4 | -10 | 21 | 7.69 |
|  | L Superior Temporal Gyrus/ Area Id1 | -40 | -14 | -8 | 28 | 8.18 |
|  | L Rolandic Operculum | -48 | 0 | 6 | 143 | 7.9 |
|  | L Insula Lobe | -32 | 24 | 2 | 247 | 15.48 |
|  | R Middle Frontal Gyrus | 36 | 48 | 0 | 14 | 7.33 |
|  | R Rolandic Operculum | 58 | 4 | 6 | 23 | 7.84 |
|  | R Rolandic Operculum | 68 | 10 | 6 | 14 | 8.1 |
|  | L MCC | 0 | 18 | 32 | 1349 | 15.13 |
|  | L SupraMarginal Gyrus/ Area PFop (IPL) | -60 | -30 | 26 | 373 | 16.44 |
|  | R SupraMarginal Gyrus/ Area PF (IPL) | 60 | -36 | 28 | 187 | 10.82 |
|  | R Middle Frontal Gyrus | 36 | 40 | 26 | 52 | 9.1 |
|  | R MCC | 6 | -20 | 34 | 89 | 9.73 |
|  | L MCC | -4 | -12 | 36 | 20 | 8.5 |
|  | RPrecentral Gyrus | 44 | -4 | 40 | 10 | 7.63 |
|  | R Middle Frontal Gyrus | 46 | 4 | 52 | 30 | 8.4 |
|  | R Superior Medial Gyrus | 4 | 26 | 56 | 43 | 9.26 |
| *Negative* | R Cerebelum IX | 2 | -60 | -48 | 10 | 7.58 |
|  | R Cerebelum Crus 2 | 18 | -86 | -44 | 19 | 8.56 |
|  | R Cerebelum Crus 2 | 38 | -62 | -44 | 13 | 8.01 |
|  | R Cerebelum Crus 2 | 28 | -86 | -40 | 41 | 8.15 |
|  | R Medial Temporal Pole | 36 | 8 | -36 | 22 | 7.78 |
|  | L Medial Temporal Pole | -26 | 14 | -36 | 94 | 10.7 |
|  | L Inferior Temporal Gyrus | -40 | 10 | -36 | 26 | 7.95 |
|  | R Inferior Temporal Gyrus | 52 | -4 | -32 | 64 | 10.15 |
|  | R Cerebelum Crus 2 | 22 | -90 | -32 | 16 | 7.64 |
|  | R Cerebelum Crus 1 | 32 | -80 | -30 | 18 | 8.31 |
|  | R Lingual Gyrus/ Area hOc1 [V1] | 4 | -68 | 4 | 7731 | 24.5 |
|  | L Rectal Gyrus (VMPFC/ mOFC) | -2 | 38 | -18 | 429 | 12.95 |
|  | R ParaHippocampal Gyrus/ CA3 (Hippocampus) | 22 | -16 | -20 | 94 | 13.51 |
|  | R ParaHippocampal Gyrus/ Subiculum | 16 | -6 | -24 | 31 | 9.33 |
|  | R Middle Temporal Gyrus | 62 | -10 | -18 | 83 | 9.77 |
|  | L Middle Temporal Gyrus | -60 | -12 | -22 | 19 | 9.47 |
|  | R IFG p. Orbitalis (latOFC) | 36 | 34 | -14 | 43 | 8.19 |
|  | L Inferior Temporal Gyrus | -48 | -50 | -18 | 24 | 17.7 |
|  | L IFG p. Orbitalis | -36 | 30 | -16 | 23 | 7.36 |
|  | R Hippocampus | 38 | -20 | -14 | 12 | 7.62 |
|  | R Amygdala (CM) | 26 | 0 | -14 | 10 | 7.76 |
|  | L Middle Orbital Gyrus | -44 | 48 | -10 | 21 | 12.31 |
|  | L Middle Temporal Gyrus/ Area TE 3 | -64 | -10 | -6 | 56 | 13.83 |
|  | L IFG p. Orbitalis | -48 | 30 | -10 | 16 | 16.94 |
|  | L Mid Orbital Gyrus/ Area Fp1 (MPFC) | -2 | 68 | -8 | 17 | 7.42 |
|  | L Middle Frontal Gyrus (latPFC) | -38 | 46 | 0 | 11 | 7.8 |
|  | R Postcentral Gyrus/ Area 3b | 60 | -8 | 20 | 634 | 18.32 |
|  | L Superior Medial Gyrus/ Area Fp2 (MPFC) | -6 | 62 | 8 | 481 | 16.33 |
|  | R Putamen | 32 | -8 | 6 | 38 | 7.41 |
|  | L Insula Lobe | -36 | -8 | 8 | 38 | 8.31 |
|  | L Middle Frontal Gyrus | -30 | 50 | 6 | 20 | 8.47 |
|  | L IFG p. Triangularis/ Area 45 | -52 | 26 | 16 | 147 | 10.79 |
|  | L Superior Occipital Gyrus/ Area hOc2 [V2] | -6 | -102 | 10 | 10 | 8.42 |
|  | L Postcentral Gyrus/ Area 4p | -56 | -10 | 32 | 303 | 17.19 |
|  | R Cuneus/ Area hOc3d [V3d] | 12 | -94 | 20 | 72 | 7.89 |
|  | R Caudate Nucleus | 18 | 4 | 20 | 13 | 7.95 |
|  | R IFG p. Opercularis | 42 | 8 | 24 | 24 | 8.86 |
|  | R Angular Gyrus/ Area PGp (IPL) | 46 | -62 | 36 | 31 | 9.11 |
|  | L Middle Frontal Gyrus (DMPFC) | -28 | 26 | 56 | 512 | 12.69 |
|  | Area hIP1 IPS | -36 | -38 | 36 | 62 | 10.32 |
|  | R Postcentral Gyrus | 28 | -36 | 44 | 19 | 8.93 |
|  | R Postcentral Gyrus | 48 | -18 | 50 | 339 | 12.92 |
|  | L Middle Frontal Gyrus (DMPFC) | -22 | 14 | 44 | 50 | 9.04 |
|  | R Superior Frontal Gyrus (DMPFC) | 16 | 36 | 46 | 13 | 9.16 |
|  | L Inferior Parietal Lobule / Area PF (IPL) | -50 | -38 | 50 | 14 | 7.76 |
|  | R Superior Frontal Gyrus (DMPFC) | 30 | 26 | 54 | 95 | 10.06 |

^a^ This table presents brain regions whose differential responses (CS+ vs CS-) reversed immediately upon instruction. Analyses include the entire Instructed Group (n = 30). Results are whole-brain FDR-corrected (q < .05) and clusters are defined based on contiguity with voxels at uncorrected p < .001 and p < .01.
