# Supplementary material for: Instructed knowledge shapes feedback-driven aversive learning in striatum and orbitofrontal cortex, but not the amygdala
Source: eLife. 2016 May 12;5:e15192. doi: 10.7554/eLife.15192 (PMC4907691; doi:10.7554/eLife.15192)
Supplement: Figure 5—figure supplement 2—source data 1. — This table presents brain regions whose differential responses (CS+ vs CS-) did not reverse upon instruction in Instructed Group learners (n = 20). Regions that were positive in this contrast show greater activation to the pre-instruction CS+ relative to the CS- both pre- and post-instruction, whereas those that are negative show deactivation to the CS+ or increases with the CS-. Results are whole-brain FDR-corrected (q < 0.05) and clusters are defined based on contiguity with voxels at uncorrected p<0.001 and p<0.01. DOI: http://dx.doi.org/10.7554/eLife.15192.024 [file elife-15192-fig5-figsupp2-data1.docx]

*Figure 5 – figure supplement 2 – Source data 1. No reversal with instructions (main effect of CS without interaction): Instructed Group Learners (n = 20) ^a^*

| **Contrast** | **Region** | **x** | **y** | **z** | **Number of voxels** | **Robust regression intercept** |
| --- | --- | --- | --- | --- | --- | --- |
| *Positive* | R Cerebelum IX | 10 | -52 | -58 | 13 | 9.04 |
|  | R Cerebelum Crus 2 | 48 | -58 | -52 | 13 | 9.13 |
|  | R Cerebelum Crus 2 | 16 | -84 | -48 | 20 | 9.06 |
|  | R Cerebelum Crus 2 | 32 | -76 | -42 | 24 | 9.39 |
|  | R Cerebelum Crus 2 | 18 | -92 | -30 | 36 | 9.89 |
|  | R Inferior Temporal Gyrus/ Area FG2 | 50 | -58 | -26 | 11 | 8.25 |
|  | R Amygdala (LB) | 20 | 2 | -22 | 13 | 8.89 |
|  | L Middle Temporal Gyrus | -54 | 2 | -20 | 12 | 8.02 |
|  | L Inferior Temporal Gyrus | -56 | -34 | -18 | 28 | 10.45 |
|  | R ParaHippocampal Gyrus/ CA1 (Hippocampus) | 34 | -22 | -20 | 14 | 8.17 |
|  | L Inferior Temporal Gyrus | -56 | -52 | -18 | 26 | 11.92 |
|  | L IFG p. Orbitalis | -44 | 26 | -14 | 77 | 12.27 |
|  | L IFG p. Orbitalis | -32 | 46 | -16 | 11 | 8.52 |
|  | R Hippocampus | 24 | -18 | -16 | 10 | 8.29 |
|  | Cerebellar Vermis 4/5 | 0 | -58 | -8 | 48 | 9.98 |
|  | L Middle Temporal Gyrus | -60 | -56 | -2 | 88 | 14.19 |
|  | L Superior Medial Gyrus/ Area Fp1 (MPFC) | -12 | 64 | 4 | 23 | 9.49 |
|  | L Middle Temporal Gyrus | -60 | -54 | 10 | 18 | 10.31 |
|  | L Superior Medial Gyrus | -4 | 46 | 20 | 83 | 14.26 |
|  | R Precuneus | 6 | -50 | 22 | 82 | 8.51 |
|  | L Precuneus | -12 | -54 | 24 | 23 | 16.94 |
|  | L Caudate | -12 | -2 | 24 | 18 | 8.48 |
|  | R Superior Medial Gyrus (DMPFC) | 6 | 54 | 28 | 29 | 11.21 |
|  | L Superior Frontal Gyrus (DMPFC) | -16 | 54 | 30 | 27 | 8.37 |
|  | L Superior Medial Gyrus (DMPFC) | -2 | 50 | 34 | 48 | 8.97 |
|  | R Middle Frontal Gyrus (DMPFC) | 22 | 24 | 40 | 16 | 7.97 |
|  | R Middle Frontal Gyrus (DLPFC) | 44 | 16 | 44 | 13 | 7.99 |
|  | L Middle Frontal Gyrus (DLPFC) | -32 | 22 | 50 | 22 | 9.58 |
| *Negative* | L Inferior Temporal Gyrus | -36 | 2 | -36 | 15 | 9.97 |
|  | R Calcarine Gyrus/ Area hOc1 [V1] | 8 | -78 | 2 | 6949 | 21.92 |
|  | L Olfactory cortex (sgACC) | -10 | 10 | -14 | 11 | 8.98 |
|  | L Lingual Gyrus | -18 | -48 | -8 | 45 | 10.77 |
|  | L Thalamus | -20 | -30 | 0 | 61 | 14.22 |
|  | L Putamen | -22 | 16 | -2 | 22 | 8.97 |
|  | R Thalamus | 20 | -28 | 2 | 60 | 10.96 |
|  | R Middle Temporal Gyrus | 50 | -54 | 6 | 35 | 9.91 |
|  | L Postcentral Gyrus | -62 | -6 | 24 | 109 | 8.82 |
|  | R Postcentral Gyrus | 60 | -2 | 18 | 77 | 12.5 |
|  | L Superior Occipital Gyrus | -26 | -74 | 22 | 64 | 15.43 |
|  | L Postcentral Gyrus/ Area 1 | -62 | -14 | 34 | 21 | 11.91 |
|  | L MCC | -6 | 18 | 34 | 11 | 7.86 |
|  | RPrecentral Gyrus/ Area 4p | 40 | -16 | 36 | 12 | 8.26 |
|  | R Posterior-Medial Frontal | 4 | 10 | 56 | 53 | 12.17 |

^a^ This table presents brain regions whose differential responses (CS+ vs CS-) did not reverse upon instruction in Instructed Group learners (n = 20). Regions that were positive in this contrast show greater activation to the pre-instruction CS+ relative to the CS- both pre- and post-instruction, whereas those that are negative show deactivation to the CS+ or increases with the CS-. Results are whole-brain FDR-corrected (q < .05) and clusters are defined based on contiguity with voxels at uncorrected p < .001 and p < .01.
