# Supplementary material for: Instructed knowledge shapes feedback-driven aversive learning in striatum and orbitofrontal cortex, but not the amygdala
Source: eLife. 2016 May 12;5:e15192. doi: 10.7554/eLife.15192 (PMC4907691; doi:10.7554/eLife.15192)
Supplement: Figure 5—figure supplement 2—source data 2. — This table presents brain regions whose differential responses (CS+ vs CS-) did not reverse upon instruction in the entire Instructed Group (n = 30). Results are whole-brain FDR-corrected (q < 0.05) and clusters are defined based on contiguity with voxels at uncorrected p<0.001 and p<0.01. DOI: http://dx.doi.org/10.7554/eLife.15192.025 [file elife-15192-fig5-figsupp2-data2.docx]

*Figure 5 – figure supplement 2 – Source data 2. No reversal with instructions (main effect of CS without interaction): Entire Instructed Group (n = 30)* ^a^

| **Contrast** | **Region** | **x** | **y** | **z** | **Number of voxels** | **Robust regression intercept** |
| --- | --- | --- | --- | --- | --- | --- |
| *Positive* | R Cerebelum IX | 10 | -50 | -58 | 33 | 14.53 |
|  | R Cerebelum Crus 2 | 36 | -74 | -48 | 66 | 10.28 |
|  | L Cerebelum Crus 2 | -42 | -66 | -52 | 17 | 8.76 |
|  | L Cerebelum IX | -2 | -56 | -54 | 11 | 7.1 |
|  | Midbrain | -6 | -34 | -54 | 17 | 7.21 |
|  | R Cerebelum Crus 2 | 52 | -66 | -46 | 43 | 15.96 |
|  | R Cerebelum VIII | 38 | -46 | -48 | 15 | 9.71 |
|  | L Cerebelum IX | -10 | -46 | -46 | 18 | 7.82 |
|  | L Cerebelum Crus 2 | -12 | -82 | -38 | 24 | 8.3 |
|  | R Cerebelum Crus 2 | 16 | -78 | -38 | 13 | 6.88 |
|  | L Cerebelum Crus 1 | -50 | -70 | -32 | 54 | 8.82 |
|  | R Cerebelum IV-V | 32 | -30 | -34 | 11 | 6.97 |
|  | R Cerebelum Crus 1 | 40 | -58 | -30 | 13 | 7.16 |
|  | R Cerebelum Crus 1 | 38 | -74 | -30 | 12 | 7.92 |
|  | L Cerebelum Crus 1 | -14 | -80 | -28 | 28 | 9.29 |
|  | Pons | 0 | -12 | -26 | 10 | 9.41 |
|  | L ParaHippocampal Gyrus/ Subiculum | -24 | -16 | -24 | 17 | 9.37 |
|  | L Middle Temporal Gyrus | -54 | 8 | -20 | 18 | 7.73 |
|  | L Inferior Temporal Gyrus | -56 | -52 | -18 | 32 | 10.28 |
|  | L IFG p. Orbitalis (latOFC) | -32 | 46 | -18 | 29 | 7.51 |
|  | L Middle Temporal Gyrus | -68 | -34 | -16 | 17 | 7.54 |
|  | L Insula Lobe | -28 | 14 | -16 | 12 | 7.53 |
|  | R Hippocampus/ Subiculum | 26 | -22 | -14 | 39 | 7.09 |
|  | R Middle Temporal Gyrus | 56 | -24 | -10 | 15 | 6.83 |
|  | R IFG p. Orbitalis | 50 | 20 | -10 | 16 | 8.58 |
|  | L Middle Temporal Gyrus | -60 | -52 | 0 | 163 | 13.83 |
|  | L PCC | -2 | -50 | 20 | 969 | 12.98 |
|  | Cerebellar Vermis 4/5 | 4 | -50 | -4 | 15 | 7.64 |
|  | R Mid Orbital Gyrus/ Area Fp2 (MPFC) | 10 | 62 | -4 | 18 | 7.61 |
|  | L Middle Temporal Gyrus | -48 | -44 | 0 | 14 | 7.76 |
|  | R Middle Frontal Gyrus/ Area Fp1 | 32 | 58 | 2 | 11 | 9.85 |
|  | L Middle Temporal Gyrus | -60 | -32 | 2 | 34 | 7.5 |
|  | R Calcarine Gyrus | 30 | -58 | 12 | 58 | 10.34 |
|  | R Superior Temporal Gyrus | 60 | -34 | 12 | 22 | 7.32 |
|  | R Caudate Nucleus | 12 | 8 | 12 | 18 | 10.48 |
|  | L Superior Medial Gyrus (DMPFC) | 2 | 46 | 24 | 234 | 11.33 |
|  | R IFG p. Opercularis | 40 | 10 | 14 | 12 | 8.17 |
|  | L Precentral Gyrus | -48 | 4 | 18 | 25 | 9.93 |
|  | R Angular Gyrus | 44 | -48 | 22 | 41 | 7.26 |
|  | L Superior Frontal Gyrus (DMPFC) | -18 | 54 | 30 | 73 | 13.24 |
|  | L Angular Gyrus/ Area PGa (IPL) | -44 | -62 | 36 | 73 | 8.14 |
|  | L PCC | -4 | -38 | 30 | 10 | 7.52 |
|  | L Middle Frontal Gyrus (DLPFC) | -36 | 32 | 28 | 23 | 9.94 |
|  | R Middle Frontal Gyrus (DLPFC) | 44 | 34 | 28 | 15 | 7.66 |
|  | L Middle Frontal Gyrus (DLPFC) | -26 | 42 | 30 | 11 | 7.68 |
|  | R Precuneus | 10 | -66 | 34 | 23 | 8.08 |
|  | L MCC | -12 | -42 | 34 | 10 | 9.4 |
|  | R Middle Frontal Gyrus (DLPFC) | 40 | 22 | 38 | 59 | 12 |
|  | R SupraMarginal Gyrus | 48 | -40 | 38 | 12 | 8.28 |
|  | R MCC | 6 | -36 | 44 | 27 | 7.96 |
|  | L MCC | -4 | -6 | 38 | 27 | 10.02 |
|  | L MCC | 0 | -26 | 40 | 41 | 8.83 |
|  | L Precentral Gyrus (DLPFC) | -48 | 6 | 42 | 44 | 7.65 |
|  | R Inferior Parietal Lobule / Area PFm (IPL) | 50 | -56 | 44 | 10 | 8.42 |
|  | R Middle Frontal Gyrus (DLPFC) | 38 | 8 | 50 | 34 | 9.06 |
|  | R Middle Frontal Gyrus (DMPFC) | 28 | 40 | 46 | 12 | 7.9 |
|  | L Middle Frontal Gyrus (DMPFC) | -28 | 24 | 52 | 29 | 8.79 |
|  | R Superior Frontal Gyrus (DMPFC) | 16 | 36 | 54 | 18 | 8.64 |
|  | R Superior Frontal Gyrus | 30 | -8 | 60 | 34 | 7.17 |
|  | R Posterior-Medial Frontal (DMPFC) | 6 | 24 | 58 | 12 | 7.91 |
| *Negative* | R Lingual Gyrus/ Area hOc1 [V1] | 6 | -78 | 2 | 9802 | 24.07 |
|  | L Caudate Nucleus | -10 | 12 | -12 | 16 | 11.85 |
|  | L Fusiform Gyrus | -28 | -56 | -8 | 44 | 9.8 |
|  | L Lingual Gyrus | -14 | -48 | -8 | 13 | 7.59 |
|  | L Pallidum | -22 | 0 | -4 | 19 | 8.68 |
|  | L Putamen | -22 | 16 | -2 | 15 | 7.14 |
|  | R Thalamus | 20 | -28 | 2 | 84 | 12.29 |
|  | L Thalamus | -20 | -30 | 0 | 81 | 20.42 |
|  | R Middle Temporal Gyrus | 50 | -54 | 8 | 55 | 10.16 |
|  | L Rolandic Operculum | -64 | -2 | 12 | 25 | 8.13 |
|  | L Putamen | -24 | 8 | 12 | 15 | 6.86 |
|  | R Postcentral Gyrus | 60 | -2 | 20 | 42 | 8.59 |
|  | R Superior Occipital Gyrus | 28 | -64 | 32 | 12 | 7.85 |
|  | Area 3a | -34 | -20 | 40 | 15 | 6.78 |
|  | R Superior Occipital Gyrus | 26 | -62 | 42 | 11 | 8.09 |
|  | RPrecentral Gyrus | 56 | -4 | 48 | 15 | 7.27 |
|  | R Posterior-Medial Frontal | 4 | 10 | 54 | 68 | 11.32 |
|  | R Posterior-Medial Frontal | 2 | -4 | 60 | 12 | 6.75 |

^a^ This table presents brain regions whose differential responses (CS+ vs CS-) did not reverse upon instruction in the entire Instructed Group (n = 30). Results are whole-brain FDR-corrected (q < .05) and clusters are defined based on contiguity with voxels at uncorrected p < .001 and p < .01.
