# Supplementary material for: Instructed knowledge shapes feedback-driven aversive learning in striatum and orbitofrontal cortex, but not the amygdala
Source: eLife. 2016 May 12;5:e15192. doi: 10.7554/eLife.15192 (PMC4907691; doi:10.7554/eLife.15192)
Supplement: Figure 5—figure supplement 3—source data 1. — This table presents brain regions in which the ρ parameter correlated positively (warm) or negatively (cool) with the magnitude of the CS x Phase interaction, which indicates differential responses (CS+ vs CS-) that reverse upon instruction in Instructed Group learners (n = 20). Results are whole-brain FDR-corrected (q < 0.05) and clusters are defined based on contiguity with voxels at uncorrected p<0.001 and p<0.01. DOI: http://dx.doi.org/10.7554/eLife.15192.027 [file elife-15192-fig5-figsupp3-data1.docx]

*Figure 5 – figure supplement 3 – Source data 1. Correlation between instructed reversal parameter (ρ) and instructed reversal effects: Instructed Group Learners (n = 20)* ^a^

| **Contrast** | **Region** | **x** | **y** | **z** | **Number of voxels** | **Robust regression intercept** |
| --- | --- | --- | --- | --- | --- | --- |
| *Positive correlation* | L Cerebelum VIII | -22 | -62 | -50 | 48 | 11.85 |
|  | L Cerebelum VIII | -12 | -62 | -50 | 20 | 11.96 |
|  | L Cerebelum VIII | -36 | -48 | -48 | 29 | 7.39 |
|  | Lobule VIIb Hem | -4 | -82 | -46 | 21 | 11.87 |
|  | Pons | 0 | -26 | -44 | 12 | 10.55 |
|  | Lobule VIIa crusI Hem | -36 | -88 | -36 | 32 | 11.06 |
|  | L Cerebelum Crus 1 | -42 | -48 | -32 | 108 | 9.33 |
|  | R Cerebelum VIII | 10 | -70 | -34 | 13 | 7.49 |
|  | L Inferior Temporal Gyrus | -50 | -18 | -30 | 28 | 8.55 |
|  | L Cerebelum VI | -8 | -70 | -22 | 26 | 8.24 |
|  | L Cerebelum Crus 1 | -8 | -84 | -18 | 53 | 9.36 |
|  | R Cerebelum Crus 1 | 16 | -88 | -18 | 38 | 10.51 |
|  | R Inferior Occipital Gyrus/ Area hOc4v [V4(v)] | 42 | -86 | -12 | 27 | 9.97 |
|  | R Insula Lobe | 40 | 24 | 0 | 51 | 8.94 |
|  | L Superior Temporal Gyrus | -48 | -20 | 2 | 40 | 12.39 |
|  | R Insula Lobe | 34 | 14 | 10 | 26 | 9.44 |
|  | L Postcentral Gyrus/ Area OP4 [PV] | -50 | -14 | 16 | 39 | 8.91 |
|  | Bilateral thalamus | 4 | -6 | 14 | 197 | 10.83 |
|  | R Rolandic Operculum/ Area OP1 [SII] | 54 | -14 | 14 | 28 | 7.21 |
|  | R IFG p. Triangularis | 32 | 18 | 24 | 50 | 7.69 |
|  | L Cuneus | 2 | -72 | 24 | 28 | 8.92 |
|  | R Cuneus | 18 | -78 | 26 | 18 | 9.53 |
|  | L SupraMarginal Gyrus/ Area PFcm (IPL) | -52 | -38 | 28 | 56 | 13.81 |
|  | R ACC | 12 | 20 | 28 | 34 | 10.28 |
|  | R Middle Frontal Gyrus (DMPFC) | 28 | 50 | 30 | 11 | 7.53 |
|  | Area PFt IPL | 48 | -26 | 32 | 10 | 8.24 |
|  | L SupraMarginal Gyrus/ Area hIP2 (IPS) | -46 | -42 | 34 | 13 | 12.79 |
|  | L Precentral Gyrus (DLPFC) | -52 | 6 | 40 | 26 | 8.28 |
|  | L MCC | -2 | 8 | 42 | 114 | 11.74 |
|  | RPrecentral Gyrus | 52 | 2 | 48 | 58 | 9.68 |
|  | L MCC/ Area 5M (SPL) | -10 | -36 | 52 | 19 | 12.03 |
|  | L Superior Medial Gyrus (DMPFC) | 2 | 42 | 50 | 16 | 7.26 |
|  | L Precentral Gyrus | -46 | -14 | 58 | 111 | 12.92 |
| *Negative correlation* | R Medial Temporal Pole | 26 | 14 | -36 | 11 | 8.14 |
|  | R Inferior Temporal Gyrus/ Area FG2 | 46 | -66 | -10 | 14 | 11.61 |
|  | L Putamen | -22 | 14 | -6 | 12 | 9.63 |
|  | L Superior Medial Gyrus (MPFC) | -10 | 54 | 10 | 44 | 9.5 |
|  | R Middle Frontal Gyrus | 44 | 32 | 20 | 23 | 8.83 |

^a^ This table presents brain regions in which the ρ parameter correlated positively (warm) or negatively (cool) with the magnitude of the CS x Phase interaction, which indicates differential responses (CS+ vs CS-) that reverse upon instruction in Instructed Group learners (n = 20). Results are whole-brain FDR-corrected (q < .05) and clusters are defined based on contiguity with voxels at uncorrected p < .001 and p < .01.
