# Supplementary material for: Instructed knowledge shapes feedback-driven aversive learning in striatum and orbitofrontal cortex, but not the amygdala
Source: eLife. 2016 May 12;5:e15192. doi: 10.7554/eLife.15192 (PMC4907691; doi:10.7554/eLife.15192)
Supplement: Figure 5—figure supplement 3—source data 2. — This table presents brain regions in which the ρ parameter correlated positively (warm) or negatively (cool) with the magnitude of the sustained differential response in Instructed Group learners (n = 20). Results are whole-brain FDR-corrected (q < 0.05) and clusters are defined based on contiguity with voxels at uncorrected p<0.001 and p<0.01. DOI: http://dx.doi.org/10.7554/eLife.15192.028 [file elife-15192-fig5-figsupp3-data2.docx]

*Figure 5 – figure supplement 3 – Source data 2. Correlation between instructed reversal parameter (ρ) and continued response to previous CS+ vs CS- (no reversal effect): Instructed Group Learners (n = 20)* ^a^

| **Contrast** | **Region** | **x** | **y** | **z** | **Number of voxels** | **Robust regression intercept** |
| --- | --- | --- | --- | --- | --- | --- |
| *Positive correlation* | Brainstem (Pons) | 4 | -32 | -36 | 36 | 14.96 |
|  | L Cerebelum IX | -2 | -50 | -34 | 29 | 10.17 |
|  | R Cerebelum Crus 1 | 32 | -82 | -26 | 21 | 9.26 |
|  | R Middle Temporal Gyrus | 52 | -50 | -2 | 21 | 10.82 |
|  | R Thalamus | 16 | -10 | 0 | 46 | 10.85 |
|  | R Heschls Gyrus/ Area TE 1.0 | 48 | -20 | 6 | 32 | 10.88 |
|  | L Superior Occipital Gyrus/ Area hOc1 [V1] | -14 | -96 | 6 | 34 | 8.37 |
|  | R Calcarine Gyrus/ Area hOc1 [V1] | 14 | -74 | 8 | 10 | 8.07 |
|  | R Middle Temporal Gyrus | 52 | -66 | 12 | 19 | 14.34 |
|  | R Superior Occipital Gyrus | 24 | -86 | 20 | 56 | 8.89 |
|  | L Middle Temporal Gyrus/ Area PGp (IPL) | -48 | -70 | 18 | 12 | 9.36 |
|  | R Superior Medial Gyrus (DMPFC) | 12 | 54 | 20 | 16 | 10.52 |
|  | R Cuneus | 20 | -78 | 26 | 41 | 11.79 |
|  | L Superior Temporal Gyrus/ Area PFcm (IPL) | -56 | -44 | 22 | 16 | 8.99 |
|  | R Superior Medial Gyrus (DMPFC) | 12 | 62 | 24 | 12 | 10.11 |
|  | R SupraMarginal Gyrus/ Area PFop (IPL) | 50 | -22 | 26 | 16 | 8.72 |
|  | R Middle Occipital Gyrus/ Area PGp (IPL) | 42 | -72 | 28 | 17 | 13.58 |
|  | L Superior Occipital Gyrus | -22 | -68 | 32 | 33 | 9.53 |
|  | R MCC | 8 | -6 | 34 | 10 | 11.35 |
|  | L Middle Frontal Gyrus (DLPFC) | -40 | 28 | 46 | 33 | 11.72 |
|  | L Middle Frontal Gyrus (DMPFC) | -26 | 34 | 50 | 19 | 8.51 |
| *Negative correlation* | R Cerebelum Crus 1 | 40 | -56 | -40 | 26 | 8.79 |
|  | L Cerebelum Crus 2 | -6 | -90 | -34 | 30 | 9.59 |
|  | R Inferior Temporal Gyrus | 50 | -34 | -28 | 19 | 9.61 |
|  | L Middle Temporal Gyrus | -46 | 4 | -26 | 114 | 16.53 |
|  | L ParaHippocampal Gyrus/ Subiculum | -26 | -14 | -28 | 24 | 7.85 |
|  | R Amygdala (LB) | 34 | 2 | -28 | 11 | 12.72 |
|  | R Amygdala (SF) | 12 | -6 | -20 | 15 | 10.89 |
|  | R Amygdala (SF) | 20 | 2 | -18 | 43 | 9.54 |
|  | R Hippocampus | 24 | -14 | -16 | 32 | 9.76 |
|  | L Middle Temporal Gyrus | -66 | -54 | -6 | 36 | 11.35 |
|  | R IFG p. Triangularis (latPFC) | 52 | 44 | -2 | 27 | 7.6 |
|  | L Thalamus | -4 | -26 | 4 | 11 | 11.78 |
|  | R Paracentral Lobule | 12 | -34 | 56 | 27 | 8.52 |

^a^ This table presents brain regions in which the ρ parameter correlated positively (warm) or negatively (cool) with the magnitude of the sustained differential response in Instructed Group learners (n = 20). Results are whole-brain FDR-corrected (q < .05) and clusters are defined based on contiguity with voxels at uncorrected p < .001 and p < .01.
