# Supplementary material for: Instructed knowledge shapes feedback-driven aversive learning in striatum and orbitofrontal cortex, but not the amygdala
Source: eLife. 2016 May 12;5:e15192. doi: 10.7554/eLife.15192 (PMC4907691; doi:10.7554/eLife.15192)
Supplement: Figure 6—figure supplement 1—source data 1. — This table presents brain regions in which the DLPFC response correlated positively (warm) or negatively (cool) with the magnitude of the CS x Phase interaction, which indicates differential responses (CS+ vs CS-) that reverse upon instruction in Instructed Group learners (n = 20). Results are whole-brain FDR-corrected (q < 0.05) and clusters are defined based on contiguity with voxels at uncorrected p<0.001 and p<0.01. DOI: http://dx.doi.org/10.7554/eLife.15192.031 [file elife-15192-fig6-figsupp1-data1.docx]

*Figure 6 – figure supplement 1 – Source data 1. Correlation between dorsolateral prefrontal response to instructions and instructed reversal effect: Instructed Group Learners (n = 20)* ^a^

| **Contrast** | **Region** | **x** | **y** | **z** | **Number of voxels** | **Robust regression intercept** |
| --- | --- | --- | --- | --- | --- | --- |
| *Positive* | R Cerebelum Crus 2 | 46 | -64 | -48 | 20 | 10.15 |
|  | L Cerebelum VI | -14 | -74 | -24 | 18 | 9.01 |
|  | R Inferior Occipital Gyrus / Area hOc4la | 46 | -72 | -16 | 12 | 12.56 |
|  | R IFG p. Orbitalis | 44 | 44 | -12 | 25 | 11.19 |
|  | R Putamen | 24 | 8 | 12 | 25 | 10.04 |
|  | R Superior Medial Gyrus | 10 | 50 | 30 | 10 | 10.93 |
|  | R Inferior Parietal Lobule / Area hIP1 (IPS) | 38 | -50 | 38 | 24 | 11.19 |
|  | R Superior Frontal Gyrus | 24 | 18 | 50 | 11 | 10.55 |
| Negative | L Temporal Pole | -32 | 8 | -20 | 18 | 7.89 |
|  | L Rectal Gyrus (mOFC) | -4 | 40 | -18 | 9 | 10.03 |
|  | L IFG p. Triangularis | -56 | 38 | 0 | 29 | 10.3 |
|  | L Superior Temporal Gyrus | -52 | -20 | 4 | 28 | 9.65 |
|  | L Superior Frontal Gyrus (DMPFC) | -16 | 54 | 32 | 42 | 7.69 |
|  | L Superior Medial Gyrus (DMPFC) | -6 | 60 | 38 | 12 | 8.1 |
|  | RPrecentral Gyrus | 40 | -16 | 58 | 36 | 8.36 |

^a^ This table presents brain regions in which the DLPFC response correlated positively (warm) or negatively (cool) with the magnitude of the CS x Phase interaction, which indicates differential responses (CS+ vs CS-) that reverse upon instruction in Instructed Group learners (n = 20). Results are whole-brain FDR-corrected (q < .05) and clusters are defined based on contiguity with voxels at uncorrected p < .001 and p < .01.
